# Financing and payment reforms for primary health care and universal health care in Africa

**DOI:** 10.4102/phcfm.v14i1.3716

**Published:** 2022-10-19

**Authors:** Shabir Moosa

**Affiliations:** 1 Department of Family Medicine and Primary Care, Faculty of Health Sciences, University of the Witwatersrand, Johannesburg, South Africa; 2 Department of Family Medicine, Johannesburg Health District, Gauteng Department of Health, Johannesburg, South Africa; 3 African Forum for Primary Health Care, Johannesburg, South Africa

A Lancet Global Health Commission on financing primary health care (PHC) published its findings and recommendations recently.^1^ The lead author, Prof. Kara Hanson of the London School of Hygiene and Tropical Medicine, presented her findings at an African Forum for Primary Health Care (AfroPHC) webinar on the same topic.^2^ While the report had a good deal of technical detail, it can be somewhat difficult to read. However, the presentation was extremely succinct and useful.

Low-income countries, mostly in Africa, spent $3.00 per capita in 2018, whereas high-income countries spent $840.00. This global inequity outdoes any national-level Gini coefficient and speaks volumes for the lack of global social solidarity at the most basic of levels of PHC – how shameless and painful for a global citizen. Much of that $3.00 goes to funding infrastructure and human resources for nominal PHC services. If global ‘universal’ health coverage is to be achieved, this funding inequity must be the basis for global action to ensure resilient basic care across the world. Further contributions to low-income countries must be seen as an investment by high-income countries, especially in the light of coronavirus disease 2019 (COVID-19) and its pervasive and persistent impact on the global economy. ‘Donor’ funding of $8.00 per capita to low-income countries does not even begin to address the inequity, especially as the funding is directed at donor country priorities of verticalised programmes and creating internal brain drains of health professionals that often undermine an integrated comprehensive people-centred approach to PHC. Lower-middle-income countries do not fare much better at $16.00 per capita, with $8.00 per capita in ‘donor’ funding. Upper-middle-income countries spent $73.00 per capita on PHC (receiving $6.00 per capita in donor funding), which raises the spectre of indulgent, wasteful PHC spending amongst high-income countries. It is no wonder that health professionals are drawn to high-income countries draining such needy spaces such as PHC of human resources.^1^

This low spending is not a function of the commitment of governments in low-middle-income countries to PHC. Governments in these countries appear marginally more committed to PHC compared with high-income countries. Primary health care spending by government (as a percentage of total government expenditure on health) is almost the same, with low-income countries at 37.5%, lower-middle-income countries at 36.4% and high-income countries at 35.7%. For low-middle-income countries to achieve equity would require them to raise real spending on PHC to $523.00, which is an impossible task when total government expenditure in low- and middle-income countries sits at $40.00 and $104.00 per capita, respectively. The commission showed that higher government spending on PHC is strongly associated with better service coverage.^1^ The world needs to examine itself for its commitment to global social solidarity!

Another key finding was that whilst PHC spending by governments in low-income countries was $3.00 per capita in 2018, out-of-pocket expenditure by citizens of low-income countries was four times more, at $12.00 per capita. Low-middle-income countries are similar in distortion, with $16.00 per capita spending on PHC by governments and $23.00 out-of-pocket expenditure. Most of this spending appeared to be on medicines, a mix of fake and branded products, mostly driving profits to international pharmaceutical companies from high-income countries. It also shows the lost opportunity in low-middle income countries for governments to reroute this large out-of-pocket funding through appropriate taxation into a single pool fund that can be appropriately used across the country, based on population risk and need. This pooling is often confounded by multiple pools of health funding, corruption and a lack of population trust in government as custodian of such funds.^1^

This rearrangement based on pooling and insurance systems can lay the basis for strategic purchasing systems that separate funder (government) from purchaser (national insurance funds) from providers (health establishments), who can be contracted at a very decentralised level in best practice payment systems, as described by the commission. This allows the possibility of private providers to be brought into the equation and advance the cause of government as custodian of the entire health system, as advocated in both the World Health Report on PHC in 2008 and the Astana Declaration in 2018.^3,4^ The Strategic Purchasing: Africa Resource Centre (SPARC^1^), an organisation supporting AfroPHC,^2^ provides an extensive support system to ministries of finance and health in African countries. Strategic Purchasing: Africa Resource Centre is supported by the Joint Learning Network (JLN^3^), a global support system among low-middle-income countries.

The commission also found that capitation (mostly blended), which is extensively used in upper- and lower-middle-income countries is marginally used in low-income countries. Most of the payments were via inputs, invariably managed at a relatively centralised and unresponsive level. It also found that public PHC providers at the coalface have limited autonomy on various aspects of PHC provision, with the ability to respond to communities by choosing the mix of PHC services and hiring and firing staff being the weakest.^1^ A commentary from a PHC provider perspective on the commission’s findings stressed that blended capitation provides the best way to keep PHC funding simple and allow the latitude for teamwork, prevention-promotion and flexibility in response to community needs.^5^

The Lancet Global Commission advocates for improved revenue mobilisation (through tax revenues) and pooling (into a fund bringing in ‘aid’ and defined to cover PHC) and strong resource allocations for PHC (given the political economy and political support) to provide direct support to providers at a decentralised level. It stressed that capitation payment systems create the strongest incentives to deliver people-centred PHC, and those countries should progressively work towards a blended payment system for PHC, with capitation at its centre. The commissioners included Africans from South Africa, Rwanda, Tanzania and Sierra Leone, and the commission represents an important tool for advocacy of appropriately funded PHC for universal health coverage in Africa. All family doctors should be familiar with it.^1^

## Footnotes

1 See https://sparc.africa/

2 See https://afrophc.org/

3 See https://www.jointlearningnetwork.org/
